# Cervical Lymph Node Metastasis As the Initial Manifestation of Colorectal Cancer: Progression to Complete Clinical Response After Systemic Treatment

**DOI:** 10.7759/cureus.111061

**Published:** 2026-06-17

**Authors:** Arantxa A Corona Bacelis, Eduardo D Cruz Mendoza, Roberto Ángel Nuñez González, Aldo J Díaz Aguilar, Adriana Pasaran González

**Affiliations:** 1 General Surgery, Hospital de Especialidades Puebla, Ciudad de México, MEX; 2 General Surgery, Hospital de Especialidades Puebla, Puebla, MEX; 3 Coloproctology, Hospital Ángeles Puebla, Puebla, MEX; 4 Oncology Surgery, Hospital Ángeles Puebla, Puebla, MEX; 5 Radiology, Hospital de Especialidades Puebla, Puebla, MEX

**Keywords:** cervical lymph node metastasis, colonoscopy, colorrectal neoplasms, complete clinical respons, metastatic colorectal cancer (mcrc)

## Abstract

Colorectal cancer has well-established metastatic patterns, with the liver and lungs being the most frequent sites of dissemination. Cervical lymph node involvement as an initial manifestation is extremely rare and represents a significant diagnostic challenge. We describe the case of a 68-year-old female patient with no relevant comorbidities, who initially presented with left cervical lymphadenopathy at levels IV and V, associated with weight loss and a marked elevation of carcinoembryonic antigen. Histopathological examination of the cervical lymph node confirmed metastatic adenocarcinoma, and after extension studies and colonoscopy were performed, a primary tumor was identified in the transverse colon.

The patient began systemic treatment with a chemotherapy regimen based on capecitabine and oxaliplatin (XELOX) in combination with bevacizumab. Subsequently, after completing the induction phase, maintenance treatment with capecitabine plus bevacizumab was administered. During follow-up, the patient showed a gradual and sustained response, ultimately achieving a complete clinical response, confirmed by imaging studies, including computed tomography, colonoscopy, and positron emission tomography (PET).

This case highlights the importance of considering a gastrointestinal origin in patients with atypical cervical lymphadenopathy, as well as the impact of combined systemic treatment and maintenance therapy in achieving complete responses, even in unusual presentations of metastatic disease.

## Introduction

Colorectal cancer (CRC) is one of the leading causes of cancer incidence and mortality worldwide, with more than 1.9 million new cases per year [1]. Its metastatic spread follows relatively predictable patterns, mainly involving the liver, lungs, peritoneum, and regional lymph nodes [2].

Cervical lymph node involvement in colorectal cancer is extremely rare, particularly as an initial manifestation. In most reported cases, cervical dissemination occurs in the setting of advanced disease or recurrence, making it an unusual finding at the time of diagnosis [3]. This atypical presentation may lead to diagnostic delays due to the need to exclude more common head and neck primary tumors.

From an anatomical perspective, dissemination to cervical lymph nodes, especially in the left supraclavicular region, known as Virchow’s node, may be explained by the connection between abdominal lymphatic drainage and the thoracic duct [4]. This mechanism allows tumor cells from the gastrointestinal tract to migrate to distant nodal territories, although its occurrence in colorectal cancer is uncommon.

Clinically, colorectal cancer may be asymptomatic in early stages or present with nonspecific symptoms, contributing to delayed diagnosis [1]. The absence of digestive symptoms, as in the present case, increases diagnostic complexity and underscores the importance of a systematic approach in patients with cervical lymphadenopathy of uncertain etiology.

Regarding treatment, the management of metastatic CRC has evolved significantly with the incorporation of targeted therapies. Bevacizumab, an inhibitor of vascular endothelial growth factor (VEGF), has been shown to improve overall survival and progression-free survival when combined with chemotherapy [5]. These advances have made it possible to achieve deep and even complete responses in selected patients with metastatic disease [6].

This report describes an unusual case of colorectal cancer that initially presented with extensive cervical metastasis, without gastrointestinal symptoms, and achieved a complete clinical response after systemic treatment with chemotherapy and antiangiogenic therapy.

## Case presentation

A 68-year-old female patient, with no family history of cancer and no relevant comorbidities, began her illness eight months prior to evaluation with an increase in volume in the left side of the neck, accompanied by weight loss of approximately 3 kg, without associated gastrointestinal symptoms, including absence of hematochezia or alterations in bowel habits.

On physical examination, conglomerated lymph nodes were identified in the left hemineck at levels IV and V (Figure 1). An elevation of carcinoembryonic antigen (CEA) was documented, with an initial value of 360.7 ng/mL (reference range < 5.0 ng/m).

**Figure 1:**
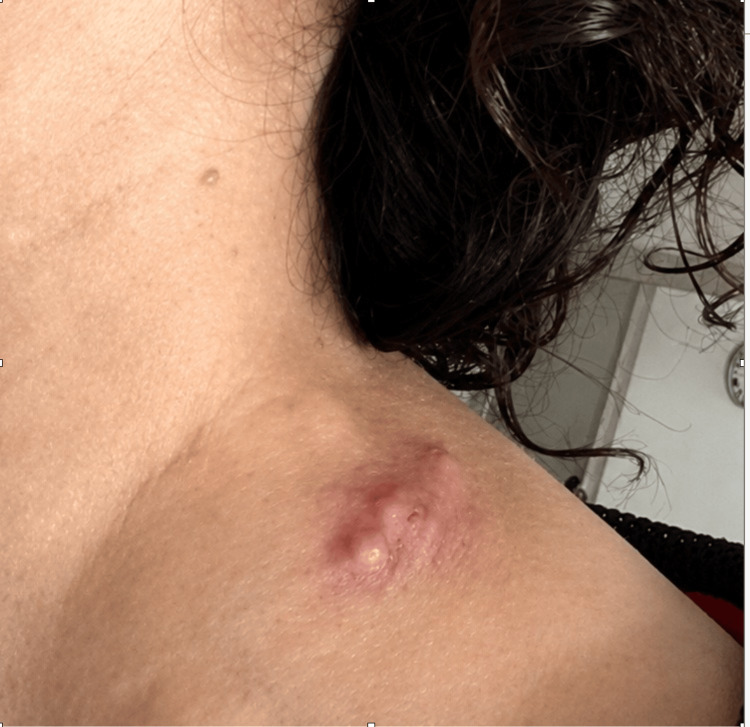
Cervical nodule Initial presentation as a left cervical lymph node conglomerate.

A contrast-enhanced thoracoabdominopelvic computed tomography scan was performed, revealing multiple lymph nodes at the cervical, mediastinal, hepatic hilum, retrocrural, para-aortic, and common iliac chain levels, with some measuring up to 8.4 cm and showing central necrosis consistent with disseminated lymph node disease (Figure 2).

**Figure 2:**
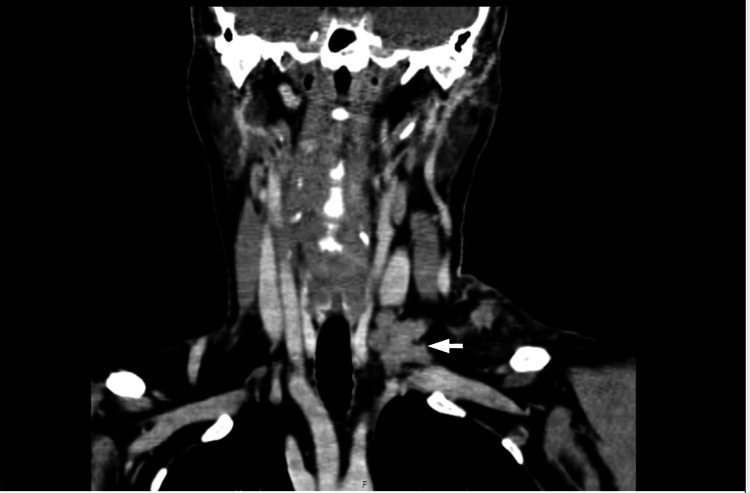
CT chest and thorax with intravenous contrast Rounded solid lymphadenopathies are identified in the left posterior cervical compartment, left supraclavicular region, and left level IV, measuring 9-10 mm. The arrow indicates the enlarged left supraclavicular/level IV lymph node.

The CT also demonstrated asymmetric wall thickening of the transverse colon, measuring 2.21 cm × 3.13 cm, which suggested a possible colorectal primary tumor (Figure 3).

**Figure 3:**
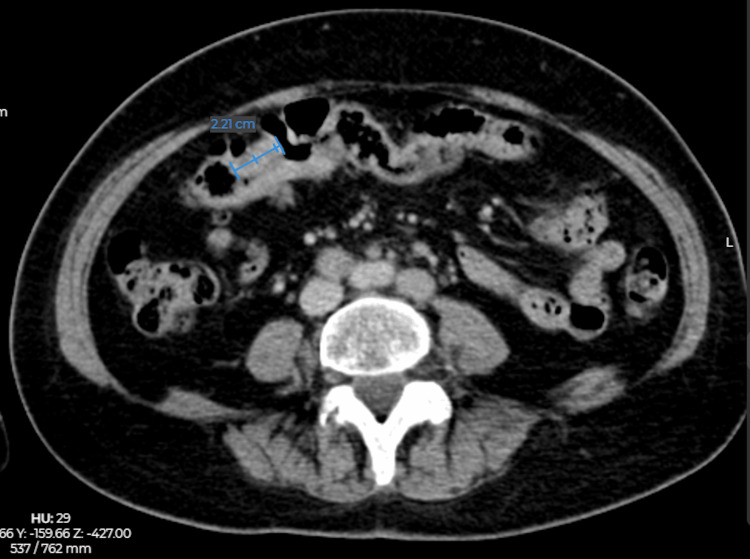
Contrast-enhanced thoracoabdominopelvic CT scan Imaging revealed asymmetric wall thickening of the transverse colon, measuring 2.21 × 3.13 cm, suggestive of a possible colorectal primary tumor.

Fine-needle aspiration biopsy (FNAB) of a cervical lymph node was performed, reporting infiltrating moderately differentiated adenocarcinoma. Subsequently, the histopathological diagnosis of infiltrating and ulcerated moderately differentiated adenocarcinoma of the colon was established (Figure 4).

**Figure 4:**
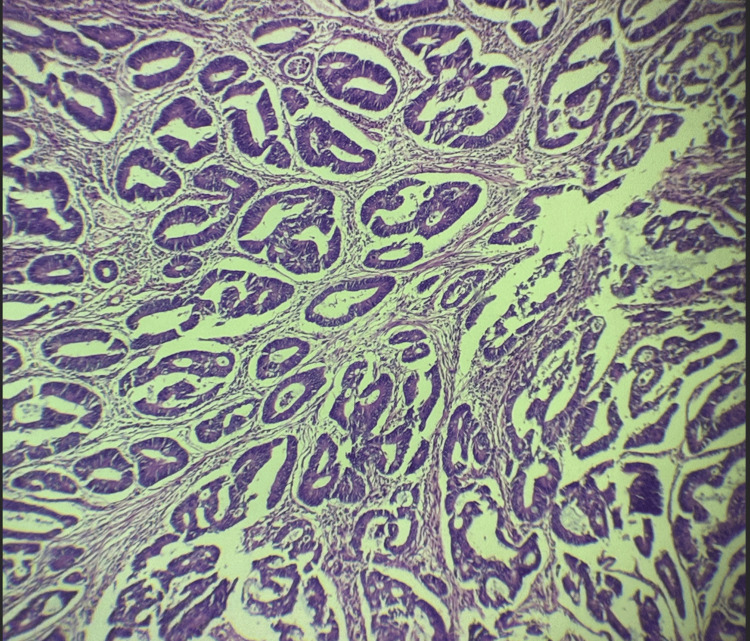
Histopathological microphotograph of conventional colorectal adenocarcinoma Hematoxylin and eosin-stained section showing an infiltrative epithelial neoplasm composed of irregular, angulated, and partially fused glands embedded in a desmoplastic stroma. These findings are consistent with moderately differentiated conventional colorectal adenocarcinoma.

Colonoscopy revealed a tumor lesion located 70 cm from the anal verge, occluding approximately 70% of the intestinal lumen (Figure 5). Based on these findings, the diagnosis of stage IV metastatic colorectal cancer (TXNXM1b) was established, with cervical, mediastinal, peritoneal, retroperitoneal, and mesenteric lymph node involvement.

**Figure 5:**
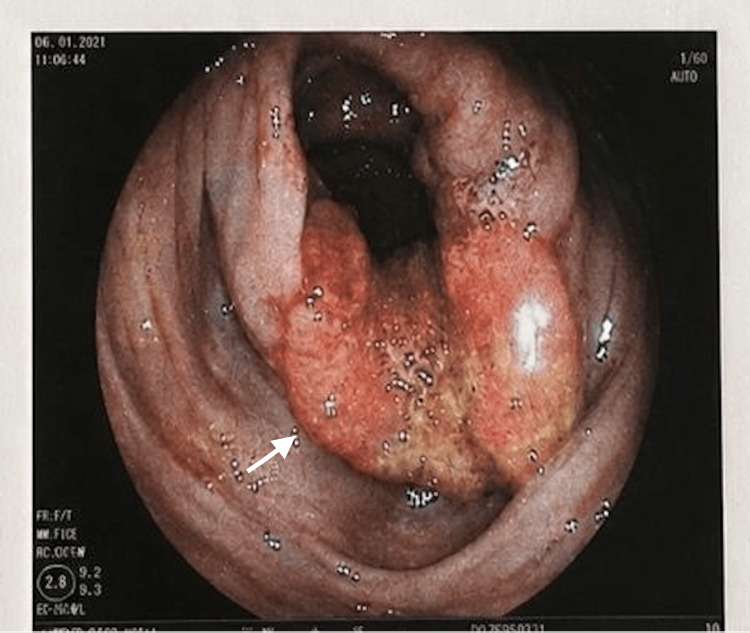
Colonoscopic image showing a colon neoplastic lesion. Colonoscopic image showing a stenosing tumoral lesion located 70 cm from the anal verge, causing approximately 70% narrowing of the colonic lumen. The arrow indicates the neoplastic lesion.

Due to the extent of metastatic nodal disease, the patient was not considered a candidate for upfront surgical management and was referred to medical oncology. Systemic therapy was selected as the primary treatment strategy with the aim of achieving disease control.

First-line treatment was initiated with XELOX, consisting of capecitabine plus oxaliplatin, in combination with bevacizumab. The patient initially received four cycles of XELOX plus two cycles of bevacizumab; however, this regimen was subsequently modified. The patient ultimately completed eight cycles of XELOX plus nine cycles of bevacizumab, followed by maintenance therapy with capecitabine and bevacizumab.

During follow-up, a significant biochemical response was documented, with a decrease in CEA to 3.42 ng/mL after the induction and maintenance phases.

After completing 13 cycles of maintenance therapy, a follow-up colonoscopy was performed on January 13, 2023, reporting colonic and rectal mucosa with no evidence of tumor activity (Figure 6).

**Figure 6:**
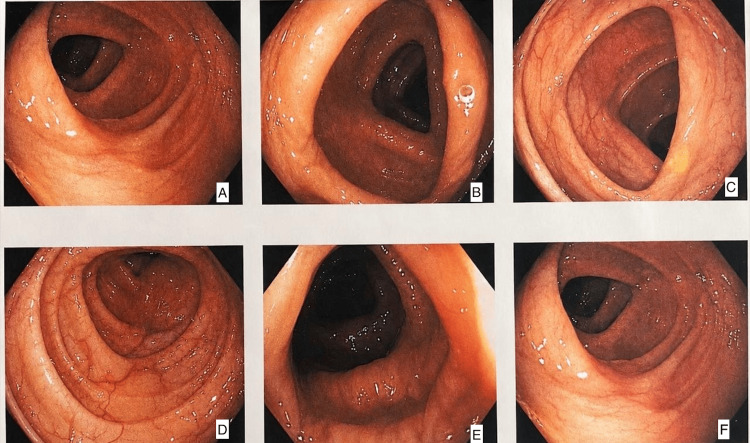
Follow-up colonoscopy after chemotherapy. Colonic and rectal mucosa without endoscopic evidence of tumoral activity, consistent with adequate response to chemotherapy. (A) Patent colonic lumen with preserved folds and no evidence of an exophytic lesion. (B) Colonic segment with adequate luminal distension; the mucosa shows a regular appearance, with no macroscopic evidence of tumoral activity. (C) Colonic mucosa with visible vascular pattern and preserved haustral folds; no lesions are identified. (D) Colon with intact mucosa, preserved superficial vascularization, and a lumen allowing passage of the colonoscope, without evidence of tumoral activity, ulceration, or bleeding. (E) Patent colonic lumen without evidence of obvious tumoral lesions, stenosis, or macroscopic infiltrative irregularity. (F) Mucosa with preserved appearance, regular folds, and adequate luminal patency. No exophytic or ulcerated lesions, nor signs of visible tumoral activity, are identified.

Subsequently, positron emission tomography (PET) showed no evidence of metabolically active neoplastic disease (Figure 7). Likewise, follow-up thoracoabdominopelvic computed tomography demonstrated no cervical lymph node enlargement (Figure 8); likewise, absence of visible tumor lesions at the level of the transverse colon, with only diffuse thickening of the sigmoid colon wall and no evidence of active tumor activity.

**Figure 7:**
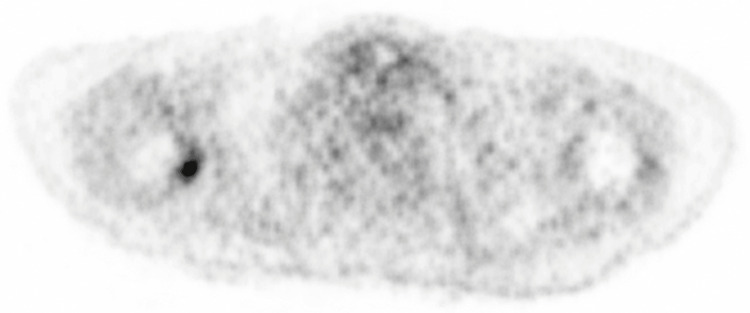
Post-chemotherapy follow-up PET/CT. The left supraclavicular region shows no evidence of pathological lymphadenopathy or abnormal fluorodeoxyglucose (FDG) uptake, consistent with an adequate metabolic response to systemic therapy.

**Figure 8:**
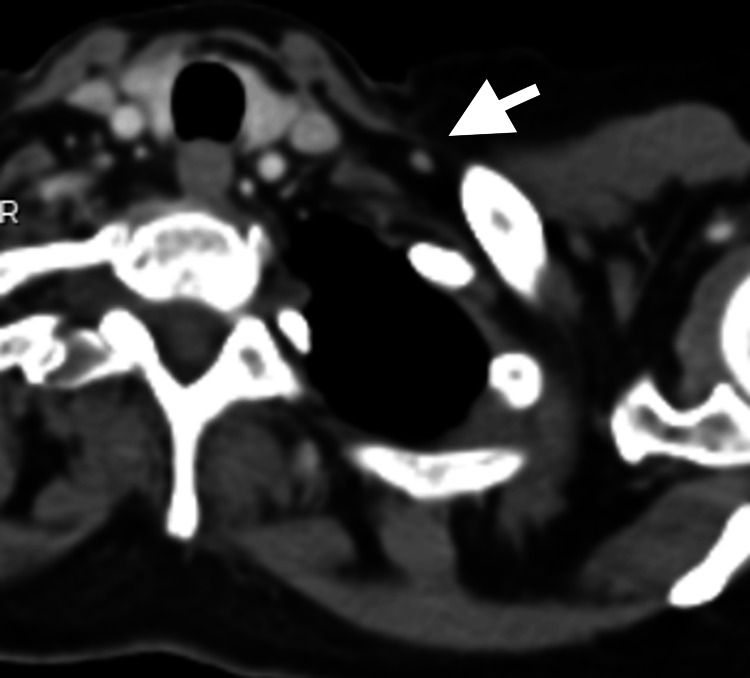
Arterial-phase contrast-enhanced CT scan of the neck and chest. The arrow indicates the left supraclavicular region, corresponding to the previous site of pathological lymphadenopathy. No residual pathological lymph node enlargement is identified.

Based on these clinical, biochemical, endoscopic, and imaging findings, a complete clinical response and disease-free period were documented, with no evidence of residual tumor activity.

## Discussion

The presentation of colorectal cancer (CRC) as an initial cervical metastasis is exceptional and represents an important diagnostic challenge. Most cases of metastatic CRC involve the liver and lungs, whereas cervical involvement is uncommon, reported in less than 1% of cases [1].

Involvement of the left supraclavicular lymph nodes is classically associated with abdominal tumors due to drainage of the thoracic duct into the venous system in this region [2]. In this context, the presence of cervical lymphadenopathy should prompt investigation for infradiaphragmatic malignancies, including colorectal cancer.

A relevant aspect of this case is the absence of gastrointestinal symptoms, which is consistent with the literature describing that colorectal cancer may remain asymptomatic, particularly in early stages, and become clinically evident only at more advanced stages [3]. This underscores the importance of considering systemic diagnoses in apparently localized presentations.

In the present case, the initial diagnosis was obtained through biopsy of the cervical lymph node. However, this presentation also highlights the importance of obtaining sufficient tissue for ancillary studies. Fine-needle aspiration may be useful as an initial diagnostic tool for suspicious cervical lymphadenopathy, but core needle biopsy may be preferable when metastatic disease of uncertain origin is suspected, as it provides more tissue for histological evaluation, immunohistochemistry, and molecular testing. This is particularly relevant in atypical metastatic patterns, where a broad immunohistochemical panel including cytokeratin 20 (CK20), caudal-type homeobox 2 (CDX2), cytokeratin 7 (CK7), special AT-rich sequence-binding protein 2 (SATB2), and molecular markers such as RAS gene family, B-Raf proto-oncogene serine/threonine kinase (BRAF), and microsatellite instability/ mismatch repair (MSI/MMR) status may improve diagnostic certainty and help guide treatment decisions.

Previous reports have highlighted the rarity of cervical or supraclavicular lymph node involvement in colorectal cancer. Wright et al. reported a 73-year-old woman with previous poorly differentiated caecal adenocarcinoma who later developed a left level IV cervical lymph node metastasis; immunohistochemistry supported intestinal origin with CK20 and CDX2 positivity, and treatment consisted of selective neck dissection followed by surveillance [7]. The patient was disease-free at 2 months, although retroperitoneal and pelvic lymphadenopathy developed during longer follow-up.

In contrast, our patient presented with extensive disseminated nodal disease rather than isolated cervical or supraclavicular involvement. Cervical lymphadenopathy was part of a broader metastatic pattern involving multiple nodal stations, which justified prioritizing systemic therapy over local treatment alone. This distinction is clinically relevant, as isolated Virchow node involvement may occasionally be the only evident distant metastatic site in upper gastrointestinal malignancies, whereas in our case, it reflected systemic lymphatic dissemination.

From a therapeutic standpoint, the management of metastatic CRC has evolved with the introduction of biological agents. Bevacizumab, a monoclonal antibody directed against vascular endothelial growth factor, has been shown to improve clinical outcomes by inhibiting tumor angiogenesis [5]. In addition, studies have shown that maintenance strategies with capecitabine and bevacizumab may prolong disease control with an acceptable toxicity profile [6].

The prognostic impact of nodal dissemination has also been described in CRC with para-aortic or apical lymph node metastases. Sahara et al. reported that para-aortic lymph node metastasis from left-sided CRC is rare and lacks a standardized treatment strategy; in their cohort, 5-year overall survival was significantly lower in patients with para-aortic nodal metastasis than in those without it [8]. Similarly, Zhou et al. found that apical lymph node metastasis was associated with worse overall survival and disease-free survival, supporting the adverse prognostic value of advanced nodal spread [9].

From a therapeutic perspective, this case also illustrates the complexity of treatment sequencing in metastatic colorectal cancer with predominant nodal dissemination. Due to the extent of metastatic disease, systemic therapy was prioritized over upfront surgical treatment. Initial treatment with XELOX plus bevacizumab achieved disease control and allowed transition to prolonged maintenance therapy with capecitabine and bevacizumab.

The complete clinical response observed in this case represents an important descriptive finding, particularly in the setting of extensive initial nodal tumor burden. This clinical course is consistent with the individualized and multimodal management described in previous reports of colorectal cancer with cervical or supraclavicular lymph node involvement. Wright et al. reported that treatment decisions in cervical nodal metastasis from colorectal cancer are generally made on a case-by-case basis and may include surgery, chemotherapy, and/or radiotherapy. They also described previously reported cases managed with neck dissection plus systemic chemotherapy or with chemotherapy plus radiotherapy, achieving short-term disease control in selected patients [7]. In contrast, our patient presented with more extensive nodal dissemination rather than isolated cervical disease, which explains the preference for systemic treatment and subsequent palliative radiotherapy to the left supraclavicular region.

This case provides additional evidence that, even in atypical presentations with extensive metastatic disease, complete responses may be achieved with appropriate systemic treatment, underscoring the importance of an individualized approach.

## Conclusions

The presentation of colorectal cancer as initial cervical lymph node metastasis is an exceptional and diagnostically challenging form of dissemination, especially in the absence of gastrointestinal symptoms. This case emphasizes the importance of considering infradiaphragmatic malignancies in the differential diagnosis of cervical lymphadenopathy and highlights the value of a comprehensive diagnostic approach, including imaging, histopathology, and immunohistochemistry.

Furthermore, this case illustrates a favorable clinical response after modern systemic therapy for metastatic colorectal cancer, including fluoropyrimidine- and oxaliplatin-based chemotherapy combined with antiangiogenic treatment such as bevacizumab, followed by maintenance therapy. However, this observation should be interpreted with caution, as it represents the outcome of a single patient and does not establish the efficacy of a specific therapeutic strategy.

The complete clinical response documented by multiple diagnostic modalities reinforces the importance of individualized treatment and close follow-up in patients with extensive nodal disease and atypical metastatic presentations. Reporting unusual cases such as this may contribute to improved clinical recognition, timely diagnosis, and better characterization of future management strategies.
